# Towards Sustainable Healthcare Risk Waste Management in South Africa: A Systematic Review of Treatment Practices and Policy Gaps

**DOI:** 10.3390/ijerph23050588

**Published:** 2026-04-30

**Authors:** Tumisang Ramodipa, Maasago Mercy Sepadi, Daniel Mmereki, Ingrid Mokgobu

**Affiliations:** Department of Environmental Health, Faculty of Sciences, Tshwane University of Technology, Staatsartillerie Rd., Pretoria 0183, South Africa; sepadimm@tut.ac.za (M.M.S.); daniel.mmereki@wits.ac.za (D.M.); mokgobumi@tut.ac.za (I.M.)

**Keywords:** healthcare risk waste, sustainability, incineration, circular economy, South Africa, policy integration

## Abstract

**Highlights:**

**Public health relevance—How does this work relate to a public health issue?**

- In South Africa, a significant knowledge gap exists in the field of healthcare waste treatment, particularly as many studies examine individual components in isolation (e.g., treatment technologies alone or facility-level compliance). This review systematically consolidates evidence across governance structures, regulatory implementation, policy gaps, infrastructural constraints, and sustainability principles within a single analytical framework.
- The review is among the first to explicitly evaluate South Africa’s healthcare risk waste practices in relation to non-burn technologies, circular economy principles, and the Sustainable Development Goals, offering a forward-looking interpretation of systemic gaps and transition pathways.

**Public health significance—Why is this work of significance to public health?**

- This study attempts to bridge a research gap and advance knowledge. Thus, the review critically assesses the current state of healthcare risk waste management in South Africa, with a specific focus on governance structures, treatment technologies, environmental and public health impacts, and alignment with sustainability principles.
- The novelty of this study lies in its integrated and policy-oriented synthesis specific to South Africa. Unlike prior studies that focus primarily on isolated treatment technologies or facility-level practices, this review systematically consolidates evidence on treatment practices, regulatory implementation, policy gaps, sustainability challenges, and governance limitations within a single analytical framework

**Public health implications—What are the key implications or messages for practitioners, policy-makers and/or researchers in public health?**

- This study can therefore complement and add information to the existing literature and help decision-makers, policy-makers and researchers to develop an efficient system to be adopted or develop any information relating to healthcare waste management.
- This will be especially helpful in a low–middle-income-country context, where documented research is not readily available and inaccessible to the general population. By situating South Africa’s experience within global LMIC patterns and incorporating environmental justice perspectives, the review broadens its relevance and expands the conceptual framing beyond technological or operational considerations.

**Abstract:**

**Background**: Effective treatment of healthcare risk waste (HCRW) is essential for preventing pathogen transmission, reducing toxic emissions, and protecting public and environmental health. South Africa faces a growing burden of HCRW driven by expanding healthcare services, population growth, and increased use of medical technologies. **Methods**: This systematic review critically examines governance frameworks, treatment technologies, and sustainability gaps in healthcare risk waste management in South Africa. The review followed Preferred Reporting Items for Systematic Reviews and Meta-Analyses (PRISMA) 2020 guidelines. Searches were conducted in PubMed, Scopus, and Web of Science for studies published between 2014 and 2025. Eighteen studies met the inclusion criteria and were analysed using a qualitative and semi-quantitative synthesis approach. **Results**: The findings indicate persistent systemic weaknesses in healthcare risk waste management. Incineration remains the dominant treatment method, reported in 72% of the included studies, and is often associated with ageing infrastructure and environmental compliance concerns. Policy fragmentation and weak regulatory enforcement were identified in 67% of studies, while 61% reported training gaps among healthcare workers and waste handlers. Poor segregation practices and illegal dumping were also frequently documented. Only 22% of studies explicitly addressed sustainability or circular economy principles, highlighting a significant policy–practice gap. **Conclusions**: Healthcare risk waste management in South Africa is therefore constrained by fragmented governance, limited infrastructure, and insufficient integration of sustainable treatment technologies. Strengthening regulatory coordination, expanding non-burn treatment technologies, and embedding circular economy principles are critical to improving environmental performance and advancing Sustainable Development Goals 3 and 12.

## 1. Introduction

Healthcare risk waste (HCRW) has increased globally over the past decade, driven by expanded access to healthcare services, rising disease burdens, population growth, and intensified use of medical technologies and personal protective equipment [1]. Effective management and treatment are essential, as inadequate handling can release hazardous pollutants and pathogens, contributing to disease transmission and long-term ecological harm [2]. Sustainable healthcare risk waste (HCRW) management refers to the safe, environmentally sound, cost-effective, and socially equitable handling, treatment, and disposal of healthcare waste throughout its lifecycle, from generation to final disposal. This approach aims to prevent infection, reduce environmental pollution, and conserve natural resources [3]. It also aligns with the United Nations Sustainable Development Goals 3 and 12 and with circular economy principles [4].

At the national level, legislation such as the National Environmental Management Act (NEMA) [5] and the National Waste Management Strategy (NWMS) [6] provides the overarching policy framework. Provincial authorities are responsible for implementation, monitoring, and enforcement; however, variations in institutional capacity and resourcing result in heterogeneous compliance across regions. At the facility level, operational practices often depend on local budgets, staff training, and contracted service providers, leading to inconsistent segregation, collection, and treatment.

These governance and operational challenges are compounded by limited treatment infrastructure nationwide. Of the authorised treatment facilities, most continue to rely on conventional incineration technologies, many of which are outdated and associated with harmful emissions, including dioxins, furans, and heavy metals [7]. The absence of a harmonised, unified national policy framework further exacerbates fragmented implementation and inconsistent enforcement across provinces [8].

When situated within the broader international and lower- and middle-income-country (LMIC) context, South Africa’s experience reflects similar systemic constraints reported elsewhere. Many LMICs face similar challenges. These include limited adoption of non-burn treatment technologies, weak regulatory capacity, inconsistent enforcement, and infrastructure deficits [9,10,11]. These constraints highlight the global need to strengthen sustainable healthcare waste systems in resource-constrained settings.

The objective of this review is to critically examine healthcare risk waste management in South Africa, with a focus on governance arrangements, treatment technologies, environmental, and alignment with sustainability principles. Specifically, the review aims to:

1. Evaluate existing policy, regulatory, and institutional frameworks and identify governance and implementation gaps.
2. Assess the treatment technologies in use and their environmental performance.
3. Analyse reported public health and ecological impacts.
4. Examine the extent to which current practices adhere to sustainability principles, including non-burning technologies, circular economy approaches, and relevant Sustainable Development Goals.

The review is informed by systems-thinking and environmental justice perspectives, recognising healthcare waste management as a socio-technical system shaped by institutional capacity, governance coherence, and socio-economic inequalities. Within South Africa, where informal recyclers and low-income communities are disproportionately exposed to environmental hazards, ineffective waste management raises significant equity and environmental justice concerns that extend beyond technical efficiency.

## 2. Methods

### 2.1. Study Design

This study adopted a systematic review design in accordance with the Preferred Reporting Items for Systematic Reviews and Meta-Analyses (PRISMA) 2020 guidelines [12]. The PRISMA checklist was used to guide the reporting of this review and is provided in the Supplementary Materials (File S1). A formal review protocol was developed prior to data extraction to enhance methodological transparency and reduce selection bias. Although the protocol was not prospectively registered in PROSPERO due to its policy-focused scope, all methodological steps, inclusion criteria, and analytical procedures were predefined and adhered to throughout the review process.

The PRISMA checklist was used to guide the planning, execution, and reporting of the review, ensuring transparency and reproducibility throughout the process. A PRISMA flow diagram summarising the identification, screening, eligibility, and inclusion of studies is provided below.

### 2.2. Eligibility Criteria

Studies were included if they: (i) focused on healthcare risk waste or hazardous medical waste; (ii) examined management practices, governance frameworks, treatment technologies, or associated environmental and health impacts; (iii) were conducted in South Africa or provided nationally relevant evidence; and (iv) were published in peer-reviewed journals or authoritative grey literature sources.

Studies were excluded if they: (i) focused solely on general municipal solid waste without specific reference to healthcare risk waste; (ii) were purely technical laboratory studies without relevance to management systems or policy; (iii) lacked sufficient methodological detail; or (iv) were not available in English.

The exclusion of non-English studies was necessary due to resource constraints; however, this language restriction may have led to the omission of relevant evidence published in other languages, which is acknowledged as a limitation of the review. To reduce publication bias and capture policy-relevant evidence, selected grey literature sources were included. These consisted of documents from government departments, regulatory agencies, and international bodies such as the World Health Organization (WHO), the Department of Forestry, Fisheries, and the Environment (DFFE), and provincial health departments. Grey literature was included only when it provided empirical data, regulatory context, or national-level insights that complemented peer-reviewed studies. Documents lacking methodological transparency, such as opinion pieces, advocacy reports, or descriptive summaries without verifiable sources, were excluded to maintain analytical rigour.

### 2.3. Search Strategy

A comprehensive literature search was conducted across three electronic databases: PubMed, Scopus, and Web of Science, selected for their extensive coverage of public health, environmental health, and waste management literature. Searches were limited to studies published between 2014 and 2025 to capture contemporary evidence reflecting recent regulatory developments and technological advancements.

Searches were limited to studies published between 2014 and 2025 to ensure the inclusion of contemporary evidence reflecting major regulatory up, policy shifts, and technological developments in healthcare risk waste management introduced over the past decade in South Africa. This time frame also aligns with significant amendments to national environmental legislation and waste management strategies implemented during this period.

The search strategy combined controlled vocabulary terms and free-text keywords related to healthcare risk waste management. Key search terms included combinations of: “healthcare risk waste”, “medical waste”, “hazardous healthcare waste”, infectious waste”, “biomedical waste”, “waste management”, “treatment technologies”, “incineration”, “non-burn technologies”, “governance”, “policy”, “environmental impacts”, and “South Africa”. Boolean operators (“AND”, “OR”) were used to refine the search.

### 2.4. Grey Literature Inclusion

To reduce publication bias and capture policy-relevant evidence, selected grey literature sources were included. These comprised reports and guidelines from government departments, regulatory authorities, and international organisations such as the World Health Organization (WHO), Department of Forestry, Fisheries, and the Environment (DFFE), and provincial health departments. Grey literature was included where it provided empirical data, regulatory context, or national-level insights not adequately represented in peer-reviewed journals. Opinion pieces and documents lacking methodological transparency were excluded to maintain analytical rigour.

### 2.5. Study Selection and Quality Assessment

The PRISMA method was applied to conduct this systematic review. PRISMA, which stands for Preferred Reporting Items for Systematic Reviews and Meta-Analyses, is an evidence-based guideline with a checklist and flow diagram to help researchers transparently and thoroughly report systematic reviews and meta-analyses, thereby improving research quality and decision-making, especially in health [12].

A systematic review is a type of research study that collects, critically evaluates, and synthesises all available evidence relevant to a clearly formulated research question, using explicit, predefined, and reproducible methods to minimise bias [12].

Therefore, a systematic review was conducted utilising the PRISMA method to determine sustainable healthcare risk waste management practices and policy gaps in South Africa.

### 2.6. Quality Appraisal Using the CASP Checklist

The methodological quality of all included studies was assessed using the Critical Appraisal Skills Programme (CASP) checklist appropriate to each study design. The CASP tool was selected for its structured, widely accepted approach to evaluating validity, methodological rigour, and relevance across qualitative and quantitative studies [13].

Each study was independently appraised against key CASP domains, including the clarity of aims, appropriate methodology, data collection, bias, and the applicability of the findings. Based on the appraisal, studies were categorised as high, moderate, or low quality. A summary of the quality appraisal outcomes is presented in the Supplementary Material (Table S2: Quality Appraisal Summary of Included Studies).

All records were screened using a two-stage process involving title/abstract review followed by full-text assessment. Screening and quality appraisal were conducted independently by four reviewers using predefined eligibility criteria. Discrepancies were resolved through discussion. When consensus could not be reached, a third reviewer was consulted. This process strengthened methodological transparency and reduced the risk of individual bias.

### 2.7. Influence of Study Quality on Interpretation

Study quality was explicitly considered during data synthesis and interpretation. Findings from high-quality studies were given greater weight when identifying dominant patterns and drawing conclusions. In contrast, results from lower-quality studies were interpreted cautiously and used primarily to highlight emerging issues or areas requiring further research. This approach ensured that the review’s conclusions were grounded in the most robust available evidence, in line with best practice for systematic reviews.

## 3. Results

### 3.1. Study Selection

A total of 2612 records were identified through database searching, including PubMed, Scopus, and Web of Science. After the removal of duplicates, 1461 records remained and were screened based on titles and abstracts. Of these, 1151 records were excluded as they did not meet the inclusion criteria.

The full texts of 149 reports were sought, and all were successfully retrieved and assessed for eligibility. Following full-text assessment, 131 studies were excluded for the following reasons: the study was not conducted in South Africa (*n* = 86), the focus was on general waste rather than healthcare risk waste (*n* = 27), or the study was not relevant to healthcare risk waste treatment technologies or management practices (*n* = 18).

**Figure 1:**
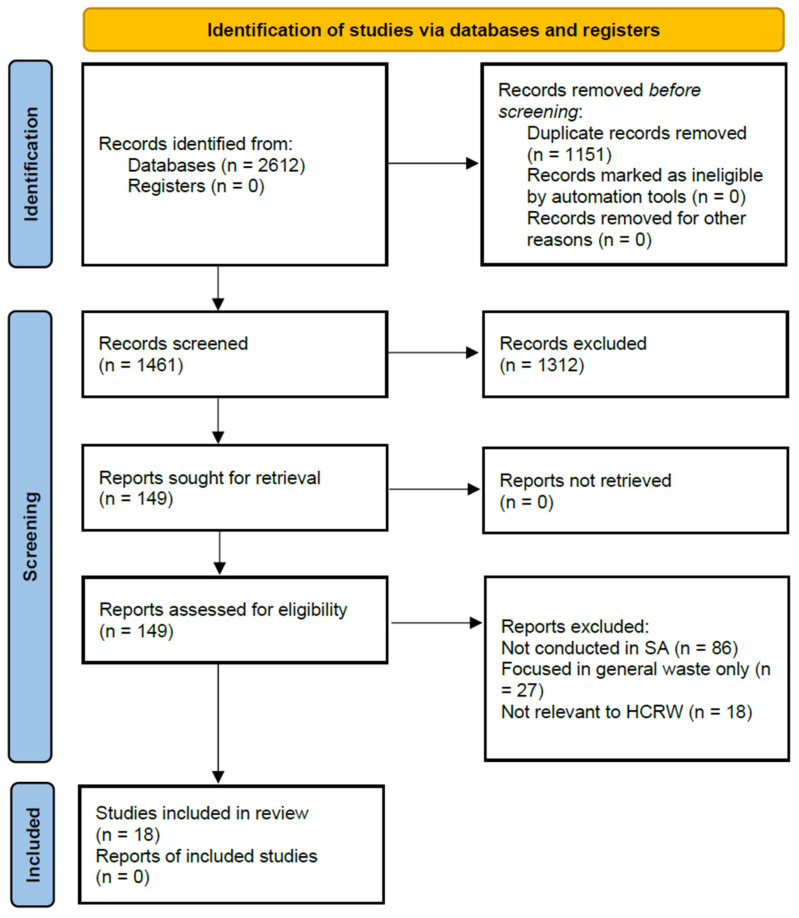
PRISMA flow diagram.

A total of 18 studies met all inclusion criteria and were included in the qualitative synthesis. Figure 1 below depicts the study selection.

### 3.2. Study Characteristics

The characteristics of the studies included in this review are summarised in Table 1, while a comprehensive data extraction matrix is provided in Supplementary Table S2. The included studies were published between 2014 and 2025 and collectively reflect healthcare risk waste management practices across multiple South African provinces, with a concentration in Gauteng, KwaZulu-Natal, and Limpopo Province.

Methodologically, the evidence base was dominated by cross-sectional and mixed-methods studies, complemented by qualitative case studies and national-level reviews. The studies were thematically classified into four primary domains: governance, treatment technologies, health and environmental impacts, and sustainability alignment.

Governance-focused studies consistently highlighted fragmented institutional responsibilities, weak regulatory enforcement, and inadequate training as key barriers to effective healthcare risk waste management. Technology-oriented studies reported a continued reliance on incineration, despite acknowledged operational, environmental, and compliance challenges, alongside limited uptake of alternative non-burning treatment options. Studies examining impacts documented elevated occupational and community health risks associated with poor waste segregation and handling practices. Sustainability-focused studies identified opportunities for waste minimisation and for integrating the circular economy. However, practical implementation remains limited. This thematic distribution directly informs the synthesis of results presented in subsequent sections. It aligns with the stated objectives of assessing governance structures, treatment technologies, impacts, and sustainability considerations within healthcare risk waste management systems in South Africa. Supplementary Table S3 illustrates the included studies [14,15,16,17,18,19,20,21,22,23,24,25,26,27,28,29,30].

### 3.3. Results Analysis and Synthesis

#### 3.3.1. Overview of Evidence Base

The 18 studies included in this review were published between 2014 and 2025, indicating a gradual increase in research attention to the management of healthcare risk waste (HCRW) in South Africa. Publication output was limited prior to 2010, followed by intermittent growth, with a noticeable increase in studies published after 2015. This trend reflects growing regulatory, environmental, and public health concerns regarding HCRW management practices.

Geographically, the studies were unevenly distributed across provinces (Table 2). Gauteng (6/18; 33%), KwaZulu-Natal (3/18; 17%) and Limpopo (2/18; 11%) were the most frequently studied provinces, while limited evidence was available from provinces such as the Western Cape, and Mpumalanga, highlighting regional research gaps. Please see Table 2 below.

In terms of methodological design (Table 3), cross-sectional studies predominated (9/18; 50%), followed by mixed-methods studies (4/18; 22%), qualitative studies (3/18; 17%), and reviews or case studies (2/18; 11%). This distribution indicates a strong reliance on descriptive observational designs, with limited longitudinal or intervention-based evidence. The predominance of cross-sectional designs limits causal inference and longitudinal understanding of improvements in HCRW management over time. The absence of intervention-based or quasi-experimental studies suggests that evidence regarding the effectiveness of policy or technological reforms remains limited.

#### 3.3.2. Primary Themes Across Studies

Table S1 presents the evidence mapping of healthcare risk waste management studies in South Africa, including the number of studies within each category and the corresponding key insights. Each study was categorised according to its dominant analytical focus: governance, treatment technologies, health and environmental impacts, or sustainability alignment. As summarised in Table 4, governance-related issues were the most frequently reported primary theme (7/18; 39%), followed by treatment technologies (5/18; 28%), impacts (4/18; 22%), and sustainability (2/18; 11%).

This thematic distribution demonstrates that while governance and technological challenges dominate the literature, sustainability-oriented analyses remain underrepresented.

#### 3.3.3. Semi-Quantitative Synthesis of Key Challenges

Although this review is qualitative, a semi-quantitative descriptive synthesis was conducted to strengthen the analytical interpretation. Table 5 summarises the frequency with which key challenges were reported across the included studies.

A reliance on incineration was reported in 13 out of 18 studies (72%), confirming incineration as the dominant HCRW treatment method in South Africa. Policy fragmentation and weak enforcement were identified in 12 studies (67%), particularly across public healthcare facilities. Training gaps among healthcare workers and waste handlers were reported in 11 studies (61%). Poor segregation at source was documented in 10 studies (56%), often linked to downstream treatment inefficiencies. Illegal dumping or improper disposal practices were reported in seven studies (39%). Integration of circular economy or sustainability principles was explicitly addressed in only four studies (22%).

These findings indicate that governance and operational weaknesses are consistently reported across the literature, while sustainable waste management approaches remain poorly embedded in practice.

#### 3.3.4. Distribution of Treatment Technologies

The distribution of HCRW treatment technologies across studies is reported. Incineration was the most frequently reported technology (13/18; 72%), followed by autoclaving or steam-based technologies (5/18; 28%). Limited evidence was found on the adoption of non-burning alternatives such as microwave treatment or chemical disinfection, which were mentioned in only three studies (17%). Several studies reported the continued use of outdated or poorly maintained incinerators, raising concerns about emissions and environmental compliance. Very few studies provided cost analyses or lifecycle economic assessments of treatment technologies. The absence of cost-effectiveness evaluations represents a critical knowledge gap, particularly in a resource-constrained, upper-middle-income-country context where financial sustainability strongly influences technology adoption decisions.

#### 3.3.5. Governance and Policy Gaps by Province

Provincial analysis reveals that governance and policy gaps were reported across all provinces represented in the literature, but were most frequently documented in Gauteng, Limpopo, and KwaZulu-Natal. Commonly cited issues included unclear institutional roles, inconsistent enforcement of national regulations, and limited monitoring of private waste service providers. Provinces with fewer studies showed insufficient evidence to draw firm conclusions, underscoring the need for geographically balanced research.

#### 3.3.6. Geographic Patterns and Setting

**Figure 2:**
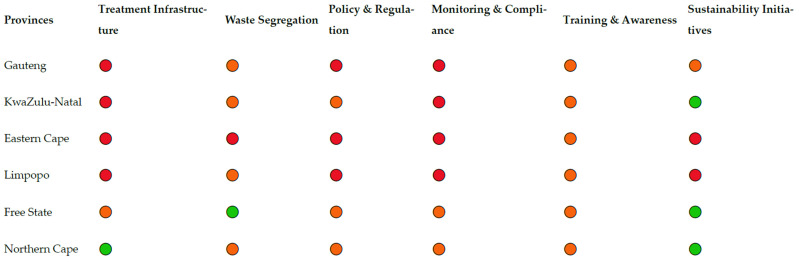
Conceptual heatmap illustrating the relative prominence of healthcare risk waste management challenges across thematic domains and provinces in South Africa. The heatmap is derived from the frequency and consistency of reported findings across included studies and represents a qualitative synthesis rather than a statistical assessment. The heatmap is presented as a qualitative interpretive synthesis to illustrate thematic distribution and prominence across studies (Figure S1: Conceptual Heatmap Development); it should not be interpreted as a quantitative or statistically weighted analysis. Note: 🔴 = Major challenge; 🟠 = Moderate challenge; 🟢 = Effective/improving area.

Figure 2 shows a conceptual heatmap illustrating the distribution of key themes in HCRW management across South African provinces.

#### 3.3.7. Conceptual Heatmap Development

To support comparative interpretation, a conceptual heatmap was developed to visualise the relative prominence of reported challenges across themes and provinces based on study frequency and consistency. The heatmap presented in Figure 2 represents a conceptual synthesis of the evidence rather than a statistical model. It was developed to visually summarise the frequency and consistency of the reporting of specific challenges across the included studies.

Two criteria informed the classification of challenges as high, moderate, or effective: (i) the proportion of studies reporting a given challenge, and (ii) the consistency of reporting across different provinces and study designs.

Challenges were classified as high when reported in the majority of included studies (≥60%) and consistently identified across multiple provinces. Moderate challenges were those reported in approximately one-third to one-half of the studies (30–59%) or in studies where findings varied by context. Areas classified as effective reflect domains where challenges were infrequently reported (<30%) or where studies documented comparatively better performance or emerging good practice.

The heatmap, therefore, serves as an interpretive visual tool to support narrative synthesis and pattern recognition, rather than as a quantitative ranking or performance score.

### 3.4. Quality Appraisal

Using the CASP, we noted a heterogeneity in the study quality typical of cross-sectional designs: variable sampling frames, limited adjustment for confounding, and a reliance on self-reported practices. Grey literature sources (e.g., industry reports) provided context but were treated with caution. Overall, the evidence base supports descriptive synthesis; meta-analysis was not feasible due to the study’s heterogeneity in design, outcomes, and reporting metrics.

## 4. Discussion

This systematic review synthesised evidence from eighteen studies examining healthcare risk waste (HCRW) management in South Africa, with particular emphasis on governance, treatment technologies, health and environmental impacts, and sustainability alignment. The findings reveal persistent structural and operational weaknesses that continue to undermine effective and sustainable HCRW management.

### 4.1. Dominance of Governance and Policy Challenges

The findings of this review highlight the need for more coherent and integrated policy reform to address the systemic fragmentation currently characterising healthcare risk waste management in South Africa. Strengthening regulatory coherence is essential. This requires better alignment between national environmental legislation, health sector regulations, and provincial enforcement mechanisms. A clearer delineation of institutional roles and the development of a unified national HCRW policy framework would help reduce duplication, close oversight gaps, and support consistent implementation across provinces. Governance-related issues emerged as the most prominent theme, reported in 39% of the included studies. More than two-thirds of studies (67%) identified policy fragmentation, weak enforcement, and unclear institutional responsibilities as key barriers to effective HCRW management. These governance failures were consistently reported across provinces, particularly in Gauteng, KwaZulu-Natal, and Limpopo, suggesting systemic rather than localised challenges. The lack of harmonised oversight between national, provincial, and facility levels limits accountability and contributes to uneven compliance with healthcare waste regulations. In South Africa, overlapping regulatory mandates, inconsistent provincial enforcement, resource constraints, and limited intersectoral coordination are key contributing factors.

### 4.2. Continued Reliance on Incineration Technologies

Given the continued dominance of outdated incineration technologies, the transition toward cleaner, non-burning treatment methods requires both regulatory and financial support. This transition requires stricter national emission standards aligned with international best practice. It also requires expanding approval pathways for non-burning technologies. Financial incentives, such as subsidised investment, public–private partnerships, or green-technology grants, could encourage facilities to adopt autoclaving, microwave treatment, and other low-emission alternatives. Strengthening technical capacity within provincial health departments and contracted service providers is similarly critical to enabling the effective operation and maintenance of these technologies. Incineration remains the dominant HCRW treatment method in South Africa, reported in 72% of the reviewed studies. While incineration is widely used for its volume-reduction capacity, many studies have highlighted operational inefficiencies, ageing infrastructure, and concerns about emissions and environmental compliance. The limited adoption of alternative non-burn technologies, such as autoclaving or microwave treatment, reflects both regulatory inertia and constrained financial and technical capacity. This technological lock-in presents a significant barrier to transitioning towards more environmentally sustainable waste treatment systems.

Comparatively, several middle-income countries have progressively shifted toward centralised non-burning technologies, supported by stricter emission standards and financial incentives for cleaner alternatives. The continued technological lock-in observed in South Africa suggests regulatory inertia and insufficient economic instruments to incentivise transition. Aligning national emission standards with international best practice could accelerate technological modernisation.

### 4.3. Operational Gaps and Health

Operational shortcomings were frequently documented, with poor segregation at source reported in 56% of studies and training gaps among healthcare workers in 61% of studies. These deficiencies increase occupational exposure risks for healthcare workers and waste handlers and contribute to inefficiencies in downstream treatment. Illegal dumping or improper disposal practices were reported in 39% of studies, raising concerns about community exposure and environmental contamination, particularly in areas surrounding healthcare facilities and waste treatment sites.

### 4.4. Limited Integration of Sustainability and Circular Economy Principles

There are also feasible, immediate entry points for integrating sustainability and circular economy principles into healthcare settings. These include waste minimisation at source through improved procurement practices, phasing out unnecessary single-use items, strengthening segregation at the facility level to enhance material recovery, and introducing pharmaceutical take-back schemes to reduce hazardous disposal burdens. Embedding lifecycle thinking within health-sector planning, together with the routine monitoring of waste volumes, treatment performance, and environmental indicators, would help ensure that sustainability considerations are operationalised rather than remaining aspirational.

Collectively, these policy and practice implications demonstrate that improving HCRW management in South Africa requires not only technological change but also institutional coordination, stronger governance mechanisms, and deliberate efforts to integrate environmental sustainability into routine healthcare operations. Despite a growing global emphasis on sustainable healthcare waste management, only 22% of the included studies explicitly addressed circular economy or sustainability principles. Where sustainability was discussed, it was largely conceptual rather than operational, with limited evidence of waste minimisation, material recovery, or resource efficiency initiatives. This gap highlights a missed opportunity to align HCRW management practices with national sustainability goals and international commitments. Furthermore, incineration-based treatment contributes to greenhouse gas emissions, linking HCRW management directly to national climate mitigation commitments. Integrating carbon accounting and lifecycle emission assessments into healthcare waste policy frameworks could strengthen alignment with South Africa’s Nationally Determined Contributions under the Paris Agreement.

### 4.5. Strengthening the Sustainability Dimension

While sustainability is central to HCRW management, the review highlights that few included studies explicitly addressed circular economy principles. To operationalise sustainability, we propose indicators across environmental (e.g., emissions reduction, safe disposal practices), economic (e.g., cost-effectiveness and efficiency of treatment technologies), and social (e.g., equitable access to safe waste management) dimensions. The findings are interpreted with consideration to WHO guidance and other international best practices, highlighting gaps in alignment, particularly in the adoption of non-burn technologies. Lifecycle considerations are discussed, comparing traditional incineration with non-burn alternatives: incineration remains energy-intensive and generates hazardous emissions, whereas non-burn technologies reduce emissions and residual waste but may require a higher initial investment and technical capacity. Finally, the findings are explicitly linked to the Sustainable Development Goals: SDG 3 (Good Health and Well-being) through reducing exposure risks to hazardous waste, and SDG 12 (Responsible Consumption and Production) through minimisation, recycling, and circular economy practices in healthcare facilities. Together, these insights provide a structured framework for advancing sustainable, policy aligned HCRW management.

### 4.6. Implications for Policy and Practice

The synthesis indicates that improving HCRW management in South Africa requires coordinated interventions across governance, technology, and capacity development. Strengthening regulatory enforcement, standardising training programmes, and incentivising the adoption of non-burn treatment technologies could significantly reduce health and environmental risks. Furthermore, embedding circular economy principles into healthcare waste policies could support long-term sustainability and system resilience. Environmental governance highlights how fragmented mandates, uneven enforcement, and weak accountability structures shape system performance and hinder coherent policy implementation. Systems resilience underscores the importance of adaptive capacity, coordinated institutions, and robust infrastructure to manage shocks such as rising waste volumes and operational failures. Environmental justice draws attention to the disproportionate exposure risks faced by low-income communities and informal waste reclaimers, emphasising equity and fairness in waste management outcomes. Together, these perspectives provide a multidimensional lens for interpreting the structural, institutional, and socio-environmental challenges identified across South Africa’s HCRW system.

#### Strategic Reform Priorities

Based on the synthesis of findings, five strategic reform priorities are proposed:

1. Development of a unified national HCRW policy integrating environmental, occupational, and public health dimensions.
2. Strengthening enforcement through improved monitoring systems and digital waste tracking mechanisms.
3. Financial incentives and public–private partnerships to accelerate the adoption of non-burn treatment technologies.
4. Standardised national training curricula for healthcare workers and waste handlers.
5. Integration of circular economy principles, including waste minimisation at source and pharmaceutical take-back schemes.

These reforms require coordinated action across national, provincial, and municipal levels to ensure sustainable and equitable waste management outcomes.

### 4.7. International Context and Transferability

When situated within a broader LMIC context, the governance, implementation, and sustainability challenges observed in South Africa reflect systemic constraints common to other LMIC settings. The findings of this review reveal substantial misalignment between current HCRW practices in South Africa and WHO best practice guidelines. The WHO recommends prioritising non-burn treatment technologies, strict emission controls, continuous monitoring, and robust segregation at source; however, most South African facilities continue to rely on ageing incinerators with limited oversight of emissions. International standards such as the WHO Blue Book guidance and Stockholm Convention recommendations emphasise minimising dioxin- and furan-emitting processes, yet these pollutants remain a documented risk in local systems. A limited adoption of non-burn technologies, weak regulatory enforcement, and inconsistent segregation practices collectively underscore gaps relative to international expectations. In Nigeria, inadequate central direction, budgetary constraints, and weak enforcement have historically undermined effective healthcare waste segregation and disposal, prompting national policy development efforts to address these gaps [9]. Similarly, studies in Ethiopia report poor segregation, informal disposal, and limited enforcement of existing guidelines, with health facilities struggling to implement WHO-recommended practices due to resource and coordination limitations [10]. Kenya’s healthcare waste management systems also face policy and infrastructure deficits, where untreated waste and poorly controlled disposal expose waste handlers and communities to increased health risks [11]. Across these LMIC contexts, structural barriers such as limited funding, weak regulatory frameworks, inadequate technical capacity, and institutional fragmentation constrain the adoption of more sustainable practices and technologies [9,10,11]. These cross-country parallels suggest that the lessons drawn from South Africa, such as prioritising regulatory coherence, integrating non-burn technology pathways, and operationalising sustainability indicators aligned with WHO guidance, may be transferable to other LMICs, particularly where similar resource constraints and governance fragmentation persist.

## 5. Conclusions

This systematic review highlights that healthcare risk waste management in South Africa is constrained by fragmented governance, inadequate infrastructure, and a limited adoption of sustainable, non-burning treatment technologies. These gaps pose significant risks to public health and the environment while impeding alignment with global sustainability frameworks, including SDG 3 and SDG 12. Strengthening regulatory coherence, expanding investment in environmentally sound treatment methods, and embedding circular economy principles are therefore critical to improving HCRW management. Without coordinated reform, healthcare risk waste management will continue to pose avoidable environmental and public health risks. A transition toward integrated governance, modernised treatment infrastructure, and sustainability-driven policy design is essential. This shift is environmentally necessary and economically and socially important for the resilience of South Africa’s health system. This review provides a robust evidence base to guide policy reforms and strategic interventions aimed at achieving environmentally sustainable and health-protective waste management systems in South Africa.

Future research should focus on assessing the environmental and economic performance of non-burning treatment technologies, assessing regulatory implementation and compliance mechanisms, and exploring innovative sustainability approaches that support environmental protection and public health in South Africa.

## Figures and Tables

**Table 1:** Frequency of studies by province.

Province	Number of Studies	Percentage
Gauteng	6	33
KwaZulu-Natal	3	17
Limpopo	2	11
Eastern Cape	1	6
Free State	1	6
Northern Cape	1	6

**Table 2:** Distribution of provinces for included studies.

Province	Number of Studies	Percentage (%)
Gauteng	6	33
KwaZulu-Natal	3	17
Limpopo	2	11
Eastern Cape	1	6
Free State	1	6
Northern Cape	1	6

**Table 3:** Methodological design of the included studies.

Study Design	Number	Percentage (%)
Cross-sectional	9	50
Mixed-methods	4	22
Qualitative	3	17
Review/case study	2	11

**Table 4:** Summarised themes for included studies.

Theme	Number	Percentage (%)
Governance	7	39
Technology	5	28
Impacts	4	22
Sustainability	2	11

**Table 5:** Reported key challenges.

Challenge	Studies Reporting	Percentage (%)
Incineration reliance	13	72
Policy fragmentation	12	67
Training gaps	11	61
Poor segregation	10	56
Illegal dumping	7	39
Circular economy integration	4	22

## Data Availability

No new data was created.

## Supplementary Materials

The following supporting information can be downloaded at: https://www.mdpi.com/article/10.3390/ijerph23050588/s1, Table S1: Evidence Mapping of Healthcare Risk Waste Management Studies in South Africa; Table S2: Quality Appraisal Summary of Included Studies; Table S3: Summary of included studies; Figure S1: Conceptual Heatmap Development; File S1: PRISMA 2020 checklist.
