# Supplementary material for: Towards Sustainable Healthcare Risk Waste Management in South Africa: A Systematic Review of Treatment Practices and Policy Gaps
Source: Int J Environ Res Public Health. 2026 Apr 30;23(5):588. doi: 10.3390/ijerph23050588 (PMC13205633; doi:10.3390/ijerph23050588)
Supplement: Supplementary file 1 [file ijerph-23-00588-s001.zip › ijerph-4150392-Supplementary Tables and Figures.pdf]

Table S1.: Evidence Mapping of Healthcare Risk Waste Management Studies in South Africa.

Policy Framework	Legislative Expectation	Reported Practice in Reviewed Studies
NEMA (South Africa)	Environmentally sound waste management and minimisation	Compliance inconsistent; enforcement weak in public facilities
National HCRW Regulations	Segregation at source and trained personnel	Poor segregation reported in 56% of studies; training gaps in 61%
Basel Convention	Environmentally sound treatment and disposal	Continued reliance on outdated incineration technologies
Stockholm Convention	Reduction of persistent organic pollutants	Emissions concerns rarely monitored or reported
Circular Economy Policy	Waste reduction and resource recovery	Explicitly addressed in only 22% of studies
	Summary and Synthesis across the included studies	

Analytical Dimension	Category	Number of Studies (n = 18)	Key Insight
Primary Theme	Governance	7 (39%)	Fragmented enforcement and unclear institutional roles dominate
	Technology	5 (28%)	Continued reliance on incineration
	Impacts	4 (22%)	Occupational and community exposure risks
	Sustainability	2 (11%)	Limited circular economy integration
Province × Dominant Challenge	Gauteng	6	Policy–practice gap; incinerator emissions

Analytical Dimension	Category	Number of Studies (n = 18)	Key Insight
	KwaZulu-Natal	3	Inconsistent regulatory enforcement
	Limpopo	2	Training gaps; poor segregation
	Eastern Cape	1	Poor waste management practices
	Northern Cape	2	Minimum treatment capacity
	Other provinces	3	Insufficient evidence
Technology × Environmental Risk	Incineration	13	Air emissions, ash disposal concerns
	Autoclaving	5	Limited capacity; segregation dependence
	Chemical disinfection	3	Chemical handling and effluent risks
	Microwave treatment	2	Minimal adoption; cost constraints

Table S2. Quality Appraisal Summary of Included Studies.

Appraisal Domain	Assessment Outcome	Confidence Level	Key Observations
Clarity of Research Aims	Most studies clearly stated objectives aligned with HCRW management issues	High	Aims were generally well articulated, particularly for governance and policy-focused studies
Study Design Appropriateness	Predominantly cross-sectional, descriptive, or case study designs	Moderate	Designs were appropriate for exploratory objectives but limited causal inference
Sampling Strategy	Often poorly justified or inadequately described	Low–Moderate	Small, convenience-based samples were common, limiting generalisability
Data Collection Methods	Methods generally described but varied in depth and rigor	Moderate	Questionnaires, interviews, and document reviews were commonly used
Data Analysis Rigor	Limited quantitative analysis and variable qualitative transparency	Low–Moderate	Few studies reported robust statistical testing or detailed qualitative analytic frameworks

Appraisal Domain	Assessment Outcome	Confidence Level	Key Observations
Consideration of Bias and Confounding	Inconsistently addressed	Low	Most studies did not explicitly discuss bias, confounders, or uncertainty
Ethical Considerations	Generally reported in peer-reviewed studies	Moderate–High	Ethical approval and consent were often mentioned, less so in grey literature
Outcome Measurement	Largely process- or perception-based rather than outcome-based	Low–Moderate	Few studies quantified environmental or health impacts
Reporting Transparency	Variable, particularly in grey literature	Moderate	Peer-reviewed studies performed better than reports and policy documents
Overall Methodological Quality	Evidence base judged as moderate	Moderate	Findings are informative but should be interpreted cautiously

Methods

Conceptual Heatmap Development

A conceptual heatmap was developed as part of the evidence synthesis to visually summarise the relative prominence of healthcare risk waste management challenges identified across the included studies. The heatmap represents a qualitative–semi-quantitative synthesis, derived from the frequency and consistency with which specific challenges were reported in the reviewed literature, rather than from measured effect sizes or statistical estimates.

Challenges were categorised as high, moderate, or low/effective based on predefined criteria. Issues classified as *high* were those reported in the majority of included studies and across multiple provinces, indicating persistent and systemic challenges. *Moderate* challenges were reported in several studies but were more context-specific or geographically limited. Challenges classified as *low or effective* were reported infrequently or described as adequately managed in most relevant studies. This approach allowed for structured comparison across governance, technological, and operational domains while remaining consistent with the qualitative nature of the systematic review.

Results: Conceptual Heatmap of Key Challenges

Figure S1 presents a conceptual heatmap summarising the relative prominence of healthcare risk waste management challenges identified across the included studies. The heatmap reflects the frequency and consistency with which specific challenges were reported in the reviewed literature, rather than measured effect sizes or quantitative outcomes.

Challenges classified as high were those reported repeatedly across a large proportion of studies and across multiple provinces, indicating persistent and systemic issues. These included reliance on incineration, governance fragmentation, illegal dumping, and inadequate training of personnel. Moderate challenges were reported in a smaller but still substantial number of studies or were context-specific, often limited to particular facility types or geographic areas. Challenges classified as low or effective were those reported infrequently or described as being adequately managed in most of the studies where they were assessed.

Overall, the heatmap highlights clear patterns in the evidence base, demonstrating that governance and regulatory weaknesses and continued dependence on outdated treatment technologies dominate the current healthcare risk waste management landscape in South Africa, while sustainability-oriented practices and circular economy approaches are reported far less frequently.

Limitations

As this heatmap is based on the frequency and consistency of reported findings rather than quantitative effect measures, it reflects patterns in the existing literature and may be influenced by reporting bias and uneven geographic or thematic coverage of studies.

Discussion: (Conceptual Framework for Healthcare Risk Waste Management Outcomes)

This review proposes a conceptual framework linking governance structures, operational practices, and health and environmental impacts in healthcare risk waste management systems.

At the governance level, regulatory frameworks, institutional arrangements, enforcement mechanisms, and resource allocation shape how healthcare facilities interpret and implement waste management requirements. Weak enforcement, fragmented responsibilities, and inadequate oversight create conditions for inconsistent compliance.

These governance conditions directly influence operational practices, including waste segregation at source, staff training, technology selection, and contractor oversight. Where governance is weak, operational practices are characterised by poor segregation, reliance on outdated incineration technologies, and limited monitoring.

Operational practices, in turn, determine health and environmental impacts. Inadequate practices increase occupational exposure risks for healthcare workers, contribute to environmental contamination through emissions and illegal dumping, and undermine broader sustainability objectives.

This framework highlights that health and environmental impacts are not isolated outcomes but are the result of upstream governance and practice failures. Effective reform therefore requires integrated interventions across all three levels rather than isolated technological solutions.

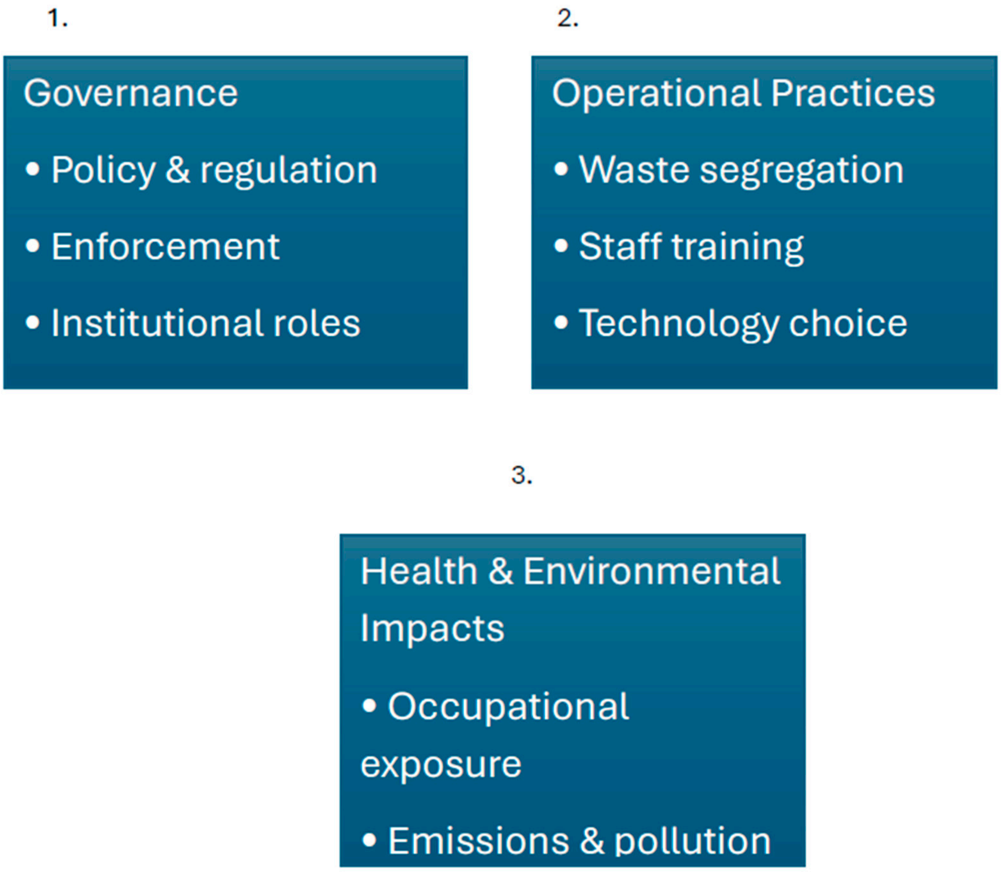

Figure S1. Conceptual Heatmap Development.

Table S3. Summary of included studies.

Author (s) (year)	Population/ Location	Study Design	Sample size	Outcome	Recommendation
**Policy & Governance**					
1. Olaniyi, F. C., Ogola, J. S., & Tshitangano, T. G. (2019) [14]	Vhembe District, Limpopo Province	Situational Mixed Method Approach	15 health care facilities	In these rural health care facilities, evidence of documented mismanagement along the waste chain: poor segregation, overfilling bins, and substandard storage.	Enforcement of healthcare risk waste guidelines, provision of standardised equipment for temporary storage, empowerment of each healthcare facility to treat at least some of the waste, and employment of non-burning techniques for treatment of waste.
2. Hlako, T. K., Morodi, T. J., Mokoena, M. M., & Molelekwa, G. F. (2025) [15]	Tshwane District Gauteng Province	Situational Mixed Method Approach	109 Private Clinics	While most (98.17%) waste is classified as infectious, there are knowledge gaps regarding treatment and disposal.	Improvements in container use and waste-handling procedures were recommended.
3. Motlatla, M., & Maluleke, T. X. (2021) [16]	Northern Cape	A descriptive, cross-sectional design.	17 hospitals	The study found that among doctors, nurses, pharmacists, and lab technicians, there was inadequate knowledge about the treatment and disposal of HCRW. Training received from professionals had minimal effect on their behaviour modification, leading to continued segregation of general waste and HCRW.	More attention should be directed behaviour, as well as towards behaviour modification. Management must ensure that HCRW-trained health professionals and HCRW management officials put into practice what they have learnt.
4. Mugivhisa, L. L., Dlamini, N.,	Academic Hospital in Tshwane,	Situational Mixed Method Approach	126 professionals	Only 69% of staff reported having received training on waste handling. Among those trained, many still engage in risky	More education on the handling of healthcare waste should be prioritised.

& Olowoyo, J. O. (2020) [17]	Gauteng province			behaviour: e.g., re-capping needles; there was no adherence to the safety practices, and risks associated with HCRW are mounting within the facility.	
5. Ramodipa, T., Engelbrecht, K., Mokgobu, I. et al. (2023) [18]	Gauteng Province	Situational analysis	42 health care facilities	There is fragmentation of responsibility: for example, HCW (healthcare waste) management is split among the National Dept of Health, the the Environmental Dept, and provincial authorities. There is limited integration of HCRW management into social, environmental, and occupational health.	Role clarity should be prioritised, including all public health aspects and environmental factors.
6. Jansen, K.E., Kocks, D.J., & Roberts, H. (2017) [19]	South Africa	Review	South Africa	Legislation is fragmented,, with the national policy on HCRW still unavailable. Key stakeholders are left unclear about their roles.	Legal requirements are Recommended for the challenge, with standardisation. Recommended for the challenge, with standardisation across the various provinces.
7. Maseko, Q.ondile. (2014) [20]	Eastern Cape Province	Qualitative method (an interpretivist paradigm).	Two selected hospitals, as well as municipal government personnel involved in waste management	HCRW is mixed with general waste and disposed of at the landfill. Waste reclaimers have access to waste which poses health and environmental risk.	The health and safety of waste reclaimers at the landfill site should be improved by ensuring good HCRW practices at the point of generation, with no evidence of HCRW at general waste landfill sites.
				**Treatment Technologies**	
8. A-Thermal (2025) [21]	South Africa	Grey literature	N/A	Treatment methods vary; the use of more sustainable technologies (e.g., non-incineration) is limited, partly due to cost, capacity, and infrastructure constraints. Older incinerators are still in use, with	Strengthening outreach campaigns, community partnerships, and patient education initiatives can empower citizens to function as watchdogs. Informed communities can put

				limited emissions control. Heavy reliance on incineration, which is environmentally harmful and alternative methods (autoclave, chemical sterilisation) are underutilized	pressure on both public and private facilities to uphold safe, compliant practices, ultimately creating a more transparent and responsible healthcare system.
9. Hangulu, L., & Akintola, O. (2017) [22]	Durban, Kwa-Zulu Natal Province	Situational qualitative approach	29 communities	At the community level, in community-based care (home-based generation of HCRW), there's poor segregation, and policymakers/stakeholders have noted illegal dumping and resource/funding constraints	With the rollout of the new primary health care model, there is a greater need to consider HCRW management in community-based care. There is a need for the Department of Health to work with the municipality to ensure that they devise measures to address improper HCRW management in the communities.
10. Zikhathile, T., Atagana, H., Bwapwa, J., & Sawtell, D. (2022) [8]	South Africa	Review	N/A	Open-pit burning of hazardous and non-hazardous waste is still an issue. Massive quantities of toxic and unpleasant gases are released into the atmosphere.	The health care industry must take steps to adopt technologies that do not rely on natural resources, including re-examining current waste management systems and evaluating HCRW treatment technologies that utilise alternative energy sources, such as solar energy.
11. Zikhathile, T., & Atagana, H. (2018) [23]	Umlazi, Kwa-Zulu Natal.	Mixed method approach	80 home-based workers.	The HCRW generated by home-based workers was found to be managed in an unsafe manner. Most of the households receiving home-based care did not have basic sanitation facilities such as toilets, running water and waste removal services,	The study recommends a new policy framework that will lead to safe management practices of generated health care risk waste to be adopted by home-based caregivers.

				aggravating the issue of HCRW mismanagement.	
12. Motlatla, M. (2015) [24]	Northern Cape	Descriptive, cross-sectional design.	17 hospitals	A lack of management support regarding HCW management, particularly because the allocation of personnel responsible for handling HCW management issues was inadequate.	Management to prioritise HCRW and ensure vital vacancies are filled where HCRW will be managed
				**Sustainable Practices and Circular Economy**	
13. Chisholm, J. M., Zamani, R., Negm, A. M., et al. (2021) [25]	Africa	Narrative review	Africa	Poor sustainable methods are observed, and the reduction of HCRW at the site where it is generated should be prioritised. Old technologies that emit hazardous pollutants are outdated and not in line with the rapid industrialisation of sustainable methods, including theoutdated and not in line with the rapid industrialisation of sustainable methods that include the circular economy.	The study suggests that awareness and understanding of proper treatment and disposal methods will reduce significant risks in the future.
14. Pululu, S.M., & Tabukeli, R. (2017) [26]	Free State Province	A cross-sectional exploratory study	A random sample of 10 hospitals was selected.	The treatment of HCRW cannot be verified or accounted for. There are no systems in place to ensure that HCRW was indeed treated accordingly.	Systems for tracking HCRW should be in place to ensure proper treatment and disposal.
				**Environmental and Health Impacts**	
15. Njoku, P. O., Edokpayi, J. N., & Odiyo, J. O. (2019)	Limpopo Province	A cross-sectional exploratory study	Population: Residents 100 m-2 km near the Thohoyandou landfill site	The community reported frequent illnesses due to the landfill's close proximity and HCRW being observed at times.	Increased monitoring of the landfill sites by officials should be prioritised with effective systems to ensure community wellness

[27]					
16. Schenck, C. J., Blaauw, P. F., Viljoen, J. M., & Swart, E. C. (2019) [28]	Durban and Tshwane (KwaZulu-Natal and Gauteng Province)	Mixed method, observational	Nine sampled landfill sites	The study revealed that HCRW was found at landfill sites. The Waste pickers were exposed to this HCRW as minimum, or no personal protection clothing was available.	Integrating waste pickers into the formal waste management system should be comprehensive to limit health risks.
17. Magagula, B. K., Rampedi, I. T., & Yessoufou, K. (2022) [29]	Johannesburg, Gauteng Province	A quantitative survey research design was adopted	A total of 371 responded to the survey.	The most common method for disposing of unused medicines was mixing them with other household waste, despite their hazardous nature.	Increasing community awareness of the proper disposal procedures for pharmaceutical waste is recommended.
18. Ngobeni, P., (2021) [30]	City of Tshwane Gauteng Province	Online questionnaire surveys use both qualitative and quantitative methods	A total of 189 community members responded	Illegal dumping of HCRW was evident within the city. The respondents were unaware of the authorities to which HCRW should be reported. The majority of the reported cases were in populated areas where health care facilities are based.	National policy should be developed with guidance to all stakeholders on how to respond to the growing illegal dumping of HCRW.
